# A deletion of *FGFR2 *creating a chimeric IIIb/IIIc exon in a child with Apert syndrome

**DOI:** 10.1186/1471-2350-12-122

**Published:** 2011-09-23

**Authors:** Aimee L Fenwick, Sarah C Bowdin, Regan EM Klatt, Andrew OM Wilkie

**Affiliations:** 1 Weatherall Institute of Molecular Medicine, John Radcliffe Hospital, Oxford, UK; 2 The Hospital for Sick Children, Toronto, Ontario, Canada

## Abstract

**Background:**

Signalling by fibroblast growth factor receptor type 2 (FGFR2) normally involves a tissue-specific alternative splice choice between two exons (IIIb and IIIc), which generates two receptor isoforms (FGFR2b and FGFR2c respectively) with differing repertoires of FGF-binding specificity. Here we describe a unique chimeric IIIb/c exon in a patient with Apert syndrome, generated by a non-allelic homologous recombination event.

**Case Presentation:**

We present a child with Apert syndrome in whom routine genetic testing had excluded the *FGFR2 *missense mutations commonly associated with this disorder. The patient was found to harbour a heterozygous 1372 bp deletion between *FGFR2 *exons IIIb and IIIc, apparently originating from recombination between 13 bp of identical DNA sequence present in both exons. The rearrangement was not present in the unaffected parents.

**Conclusions:**

Based on the known pathogenesis of Apert syndrome, the chimeric FGFR2 protein is predicted to act in a dominant gain-of-function manner. This is likely to result from its expression in mesenchymal tissues, where retention of most of the residues essential for FGFR2b binding activity would result in autocrine activation. This report adds to the repertoire of rare cases of Apert syndrome for which a pathogenesis based on atypical *FGFR2 *rearrangements can be demonstrated.

## Background

**Figure 1:**
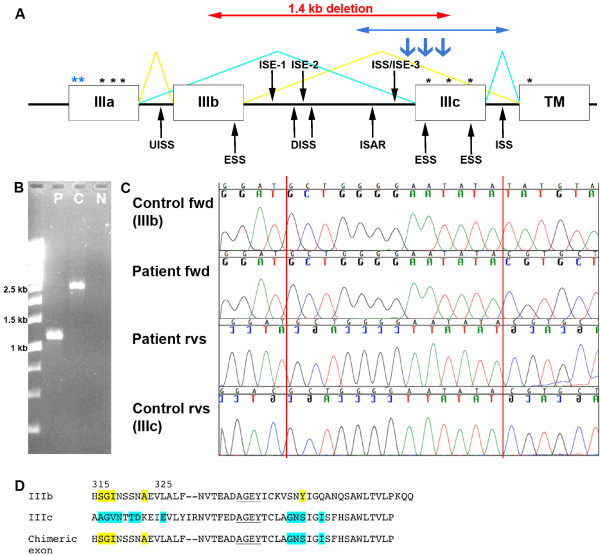
**Genome and sequence context of deletion in *FGFR2***. **A**. Schematic representation of *FGFR2 *around deleted region (red arrows); alternative splicing normally yields *FGFR2b *and *FGFR2c *spliceforms (yellow and turquoise lines respectively). Positions of previously described AS mutations are shown in blue; asterisks indicate recurrent missense mutations, downward arrows indicate *Alu *insertions, horizontal arrows represent the deletion. Other missense mutations of exons IIIa, IIIb and TM are not associated with AS (black asterisks). Intronic splicing enhancer/silencer (ISE/ISS) and exonic splicing silencer (ESS) elements are indicated with arrows (UISS - upstream intronic splicing silencer, DISS - downstream intronic splicing silencer, ISAR - intronic splicing activator and repressor). **B**. PCR of 2.5 kb region including exon IIIc, revealing a deletion in the patient (P) of ~1.4 kb compared to the control (C; N - negative control). Absence of normal-sized fragment in the patient sample is attributable to preferential amplification of the deleted allele. **C**. Sequence chromatogram of PCR products from 1B. The patient's chimeric exon is aligned with control exon IIIb and IIIc sequences. The 13 bp region of identity is shown between the red lines (fwd, forward and rvs, reverse sequence). **D**. Amino acid alignment of exons IIIb, IIIc, and the chimeric exon created by the deletion. Specific contacts for FGF10 and FGF2 are highlighted in yellow and turquoise respectively. Region of identity between exons is underlined.

**Figure 2:**
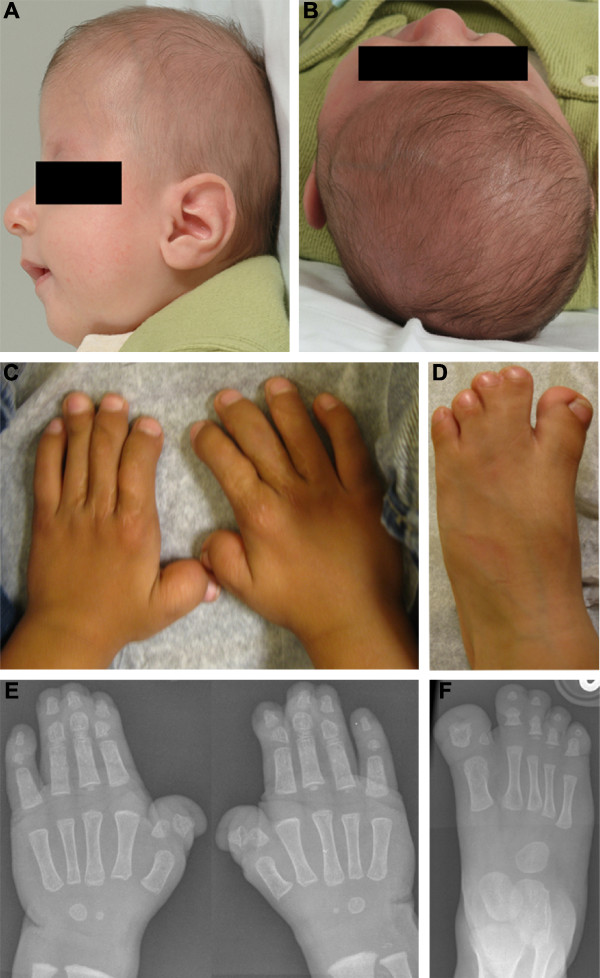
**Clinical features of Apert syndrome in the patient**. **A, B**. Note high flat skull (turribrachycephaly) associated with radiologically proven bicoronal synostosis. **C-F**. Note broad radially deviated thumbs, skin syndactyly of digits 2-4 in the hands and feet, and broad medially deviated great toes (C, post-operative after syndactyly release).

Apert syndrome (AS) is a severe malformation disorder with a birth prevalence of ~1 in 65,000, characterised by craniosynostosis (premature fusion of the cranial sutures) and bony or cutaneous syndactyly of the hands and feet [1]. Over 98% of cases are caused by one of two heterozygous mutations in exon IIIa of the fibroblast growth factor receptor 2 gene (*FGFR2*), encoding the amino acid substitutions Ser252Trp or Pro253Arg (Figure 1A; [2]). Many other pathogenic missense mutations of *FGFR2 *have been described in patients with craniosynostosis (typically with diagnoses of Crouzon, Pfeiffer or Beare-Stevenson syndromes) but these are associated with less severe abnormalities of the limbs than are present in Apert syndrome [3]. FGFR2 is one of four transmembrane FGFRs that mediate signalling downstream of fibroblast growth factor ligands (FGFs) and plays an important role in skeletal development and disease [4].

The mechanism underlying the exquisite genotype-phenotype correlation of Apert syndrome mutations (Figure 1A) needs to be understood in terms of the biology of FGF/receptor signalling and the structural pathophysiology of FGFR2 mutations [5,6]. The FGFR2 protein comprises three extracellular immunoglobulin-like domains (IgI, IgII, IgIII), a transmembrane (TM) domain and a cytoplasmic tyrosine kinase (TK) domain. Signalling occurs by trans-phosphorylation of the TK domains following dimerisation of receptor molecules mediated by binding extracellular FGF in a 2:2 complex [7]. Specificity of FGF binding is determined by the IgII and IgIII domains, and further elaborated by the mutually exclusive alternative splicing of exons IIIb and IIIc (Figure 1A) resulting in isoforms FGFR2b and FGFR2c respectively, which differ in the sequence of the second half of the IgIII domain. FGFR2b is expressed in ectoderm and interacts with specific FGFs (FGF3, FGF7, FGF10, FGF22) in the underlying mesenchyme while FGFR2c is restricted to mesenchyme and interacts with a wider repertoire of FGFs expressed in the ectoderm [8]. The narrow binding specificity of the FGFR2b isoform is largely determined by four key residues encoded near the beginning of the IIIb exon that provide specific contacts for FGF10 and related ligands [9]. The two common AS mutations (Ser252Trp and Pro253Arg) introduce additional contacts with multiple FGFs. This results both in enhanced FGFR2c signalling mediated by physiological FGFs, and in illegitimate binding by mesenchymally expressed FGFs creating an autocrine loop [10-13]; reviewed by [5,14].

In addition to the point mutations in exon IIIa, there have been three *Alu *insertions and one deletion reported as causing AS [15,16]. Although in all cases the rearrangements affect exon IIIc rather than exon IIIa, this can nevertheless be understood in terms of a shared pathophysiological mechanism with the common AS mutations. It was shown experimentally that one of these rearrangements drove illegitimate expression of the *FGFR2b *splice form in fibroblasts, a mesenchymal derivative [16]. Hence the common factor linking exon IIIa missense mutations and exon IIIc rearrangements, both causing AS, appears to be the autocrine activation of signalling by FGF10 and/or related ligands in the mesenchyme [6,14-18].

Here, we report a patient with AS caused by a variant of this latter rearrangement mechanism, in whom a 1.4 kb deletion caused by non-allelic homologous recombination between exons IIIb and IIIc has given rise to a chimeric IIIb/IIIc exon in which the reading frame is maintained.

## Case Presentation

The affected boy was the first child of healthy, non-consanguineous parents aged 24 years (mother) and 35 years (father) at the time of birth. He was born at term weighing 3.26 kg and noted to have a dysmorphic facial appearance with turribrachycephaly, open metopic suture, shallow orbits, a deviated nasal septum and high arched, non-cleft palate. Bilateral dacrocystocoeles were present. In addition he had broad radially deviated thumbs, skin syndactyly of digits 2-4 in the hands and feet, and broad medially deviated great toes (Figure 2). Three-dimensional computed tomography of the skull demonstrated bicoronal synostosis; magnetic resonance imaging of the brain showed normal intracranial structures. Cervical spine radiographs were normal, but C1 and C2 were not well visualised. Developmental progress was normal at the age of 4 years and 11 months.

A clinical diagnosis of Apert syndrome was proposed and blood obtained for genetic testing. However, two different diagnostic labs were unable to identify any pathogenic variants after DNA sequence analysis of the IIIa and IIIc exons of *FGFR2*. Further DNA sequence analysis of additional exons of *FGFR2*, and other genes in which mutations have been identified in craniosynostosis syndromes (*FGFR1*, *FGFR3 *and *TWIST1*) was also negative.

We designed a long-range PCR assay to further investigate the region around exon IIIc of *FGFR2*, looking for evidence of *Alu *insertions or other rearrangements. Amplification of a 2.5 kb region including exons IIIb and IIIc (forward primer 984 bp upstream of exon IIIb, 5'-GAATTTCAGAAGGGAACTATGGAGTAG-3', reverse primer 32 bp downstream of exon IIIc, 5'-ATAGCAGTCAACCAAGAAAAGGG-3') revealed an apparent deletion in the patient sample of ~1.4 kb compared to the control (Figure 1B). DNA sequencing of this product confirmed the presence of a 1372 bp deletion with the breakpoints occurring in a 13 bp region of perfect homology shared by the IIIb and IIIc exons (Figure 1C). This deletion results in a chimeric IIIb/IIIc exon that retains the open-reading frame and comprises the first 63 bp of exon IIIb, the 13 bp region of homology, and the final 63 bp of exon IIIc. The deletion was not present in either parental sample indicating that it had arisen *de novo *in the child. Permission was not given to determine the parental origin of the deletion.

## Discussion

The deletion identified in this patient is likely to have arisen by non-allelic homologous recombination between the 13 bp regions of identity shared by the *FGFR2 *IIIb and IIIc exons, and is expected to have two consequences. First, loss of competition between the alternative exons IIIb and IIIc may result in either constitutive splicing, or skipping, of the chimeric exon (however skipping would result in an out-of-frame translation product). Second, the chimeric exon will retain some particular IIIb or IIIc-like structural features, but lose others.

The selection of a particular exon (IIIb or IIIc) for *FGFR2 *splicing is mediated by *cis *elements either in the adjacent intron (intronic splicing enhancer/silencer: ISE/ISS) or within the exon itself (exonic splicing silencer: ESS) that bind splicing regulatory proteins to either promote or inhibit tissue-specific splicing. To date, several of these elements have been identified in the intron separating *FGFR2 *exons IIIb and IIIc, as well as one upstream of exon IIIb, one downstream of exon IIIc, and three within the exons (Figure 1A; [19]). Recently it has been shown that in the absence of tissue-specific regulators, *FGFR2c *is the default spliceform due to the stronger 3' splice site of exon IIIc and the presence of ISS and ESS elements around exon IIIb [20]. In epithelial cells, epithelial splicing regulatory proteins (ESRPs) bind to the ISE/ISS-3 element, suppressing use of the downstream exon IIIc 3' splice site and activating splicing of exon IIIb [20,21]. The deletion described here removes the entire intron between exons IIIb and IIIc, and therefore all known regulatory elements within this intron, as well as an ESS from each exon. It is difficult to predict exactly how the chimeric exon would be processed *in vivo*, but the AS phenotype of the child, which implies a gain-of-function mechanism, does indicate that production of FGFR2 containing the in-frame chimeric exon is likely to occur in critical target tissues; by contrast, skipping of both exons IIIb and IIIc would generate an isoform with loss-of-function properties [22].

The crystal structures of the FGFR2 isoforms and their ligands reveal specific contacts between receptor loops present either in FGFR2b or in FGFR2c, and the distinct FGFs with which they interact [9,23]. The deletion in the patient causes several of these contacts to be lost. However, four of five key residues for FGF10 binding are retained, making it likely that FGFR2b properties persist in the chimeric exon (Figure 1D).

## Conclusions

The case described here further demonstrates the specific mutational pathophysiology of AS, which results either from one of two missense substitutions in exon IIIa, or disruptions of exon IIIc splicing. The chimeric exon created by the deletion in this patient falls into the latter category. The encoded protein is predicted to retain FGFR2b-like binding properties, and hence lead to autocrine activation of mesenchymal signalling mediated by FGF10 or a related ligand.

## Consent

Written informed consent was obtained from the patient's parents for publication of this case report and any accompanying images. A copy of the written consent is available for review by the Editor-in-Chief of this journal.

## Competing interests

The authors declare that they have no competing interests.

## Authors' contributions

SCB and REMK undertook clinical assessment; ALF performed laboratory experiments; ALF and AOMW wrote the paper. All authors read and approved the final manuscript.

## Pre-publication history

The pre-publication history for this paper can be accessed here:

http://www.biomedcentral.com/1471-2350/12/122/prepub
